# The complete mitochondrial genome sequence of *Acentrogobius caninus* (Gobiiformes: Gobiidae)

**DOI:** 10.1080/23802359.2018.1526134

**Published:** 2018-10-29

**Authors:** Wei Shi, Min Yang, Hui Yu

**Affiliations:** a College of Life Science, Foshan University, Foshan, Guangdong, China;; b CAS Key Laboratory of Tropical Marine Bio-resources and Ecology, South China Sea Institute of Oceanology, Chinese Academy of Sciences, Guangzhou, China;; c University of Chinese Academy of Sciences, Beijing, China

**Keywords:** *Acentrogobius caninus*, complete mitochondrial genome, phylogenetic relationship

## Abstract

At present, only several species of genus *Acentrogobius* were described and information of mitochondrial DNA were also just focused on two species. Here, the complete mitochondrial genome of *Acentrogobius caninus* was completely sequenced by high throughput sequencing method. The complete mitochondrial genome was 16,614 bp in length, consisted of 13 protein-coding genes, 22 tRNA genes, 2 rRNA genes, 1 large non-coding control region, and 1 light-strand replication origin. Overall base composition values for the mitochondrial genome were 28.24, 29.20, 16.77, and 25.79% for A, C, G, and T, respectively. The gene arrangement is identical to those in typical fishes. Phylogenetic tree based on 13 protein-coding genes shows that three species of genus *Acentrogobiu* did not cluster into one clade.

The species tropical sand goby, *Acentrogobius caninus* (Gobiiformes, Gobiidae), is widely distributed throughout the Indo-west Pacific, and it lives in muddy and sandy bottoms of mangrove estuary in South China Sea and Taiwan Strait (Mao et al. 2009; Zhong 1997). Studies on *A. caninus* were seldom, only the length–weight and length–length relationship were researched (Nahar, et al., 2017). Many species of *Acentrogobius* have not been well recognized, and only species *A. pflaumii* (Jin et al. 2012) and *A. chlorostigmatoides* (Yang et al. 2016) have sequenced the complete mitogenomes. So here we described the complete mitochondrial genome of *A. caninus* and reconstructed the phylogenetic relationship of the relative species of Gobiidae and expecting for better understanding the systematic evolution of the genus *Acentrogobius* and further phylogenetic study of Gobiiformes.

The specimen was collected from Naozhou island in Zhanjiang, China (geographic coordinate: N 20°53′20.11″, E 112°28′46.20″). The specimen was preserved in ethanol and registered to the Marine Biodiversity Collection of South China Sea, Chinese Academy of Sciences, under the voucher number SW20181071708.

The complete mitochondrial genome of *A. caninus* was 16,614 bp in length (GenBank accession No. MH678615), containing 13 protein-coding genes, 22 tRNA genes, 2 rRNA genes, a control region (CR) as well as one light-strand replication origin (O_L_). The gene arrangement is identical to those in typical fishes that most of these genes are encoded by the H-stand, except for *ND6* and eight tRNA genes (tRNA*-Gln*, *-Ala*, *-Asn*, *-Cys*, *-Tyr*, *-Ser*, *-Glu*, and *-Pro*) (Boore 1999; Jin et al. 2012; Yang et al. 2016). Overall base composition values for the mitochondrial genome were 28.24, 29.20, 16.77, and 25.79% for A, C, G, and T, respectively, showing slightly AT-bias of 54.03%.

The 13 protein-coding genes encode 3803 amino acids in total. Except *COI* using GTG, the remaining 12 protein-coding genes start with ATG. Most of them use TAA or TAG as the stop codon, while *COIII* and *Cytb* use an incomplete T, *COII* and *ND4* use an unusual AGA. As in other vertebrates, the two non-coding regions, the larger one CR (977 bp) located between tRNA-*Pro* and tRNA-*Phe*, and an O_L_ located within the WANCY region.

A maximum likelihood (ML) phylogeny tree was constructed by using MEGA6 (Tamura et al. 2013), based on the sequences of 13 protein-coding genes of each mitogenome from 25 species from Gobiidae and Oxudercidae, with *Eleotris amblyopsis* and *Bostrychus sinensis* from Eleotridae as outgroup species. The ML tree showed that three species of genus *Acentrogobius* did not cluster into one clade (Figure 1). *A. caninus* and *Yongeichthys criniger* clustered together, and then joined with *A. pflaumii,* while *A. chlorostigmatoides*, *Amoya chusanensis*, and *Exyrias puntang* formed another branch.

**Figure 1.**
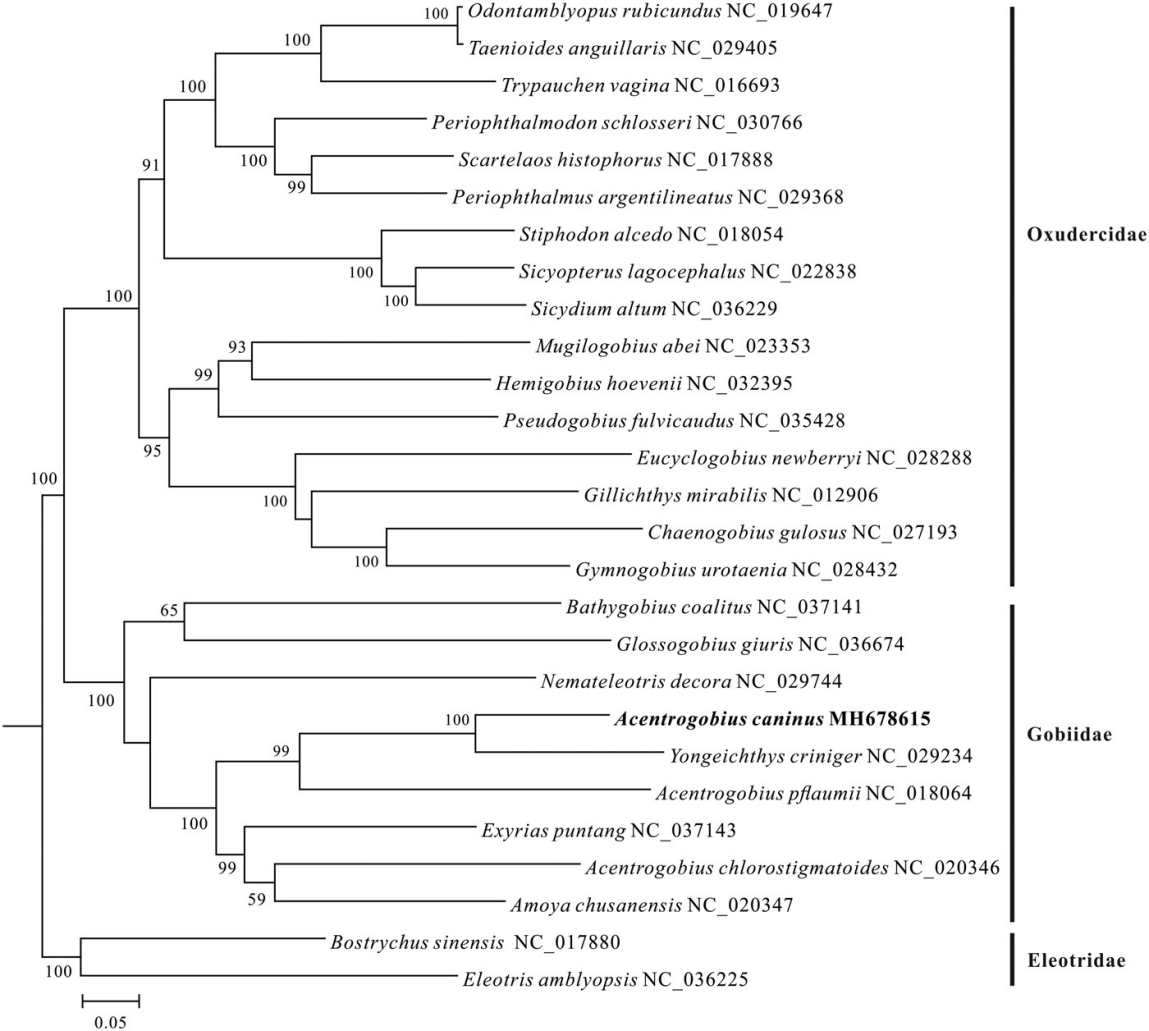
Maximum-likelihood phylogenetic tree was constructed based on the sequences of 13 protein coding genes of 27 species. The number at each node is the bootstrap probability (≥50%). The number after the species name is the GenBank accession number, and the bold species is studied in this research.
